# Gas Bubble Disease in the Brain of a Living California Sea Lion (*Zalophus californianus*)

**DOI:** 10.3389/fphys.2013.00005

**Published:** 2013-01-29

**Authors:** William Van Bonn, Sophie Dennison, Peter Cook, Andreas Fahlman

**Affiliations:** ^1^Veterinary Science Department, The Marine Mammal CenterSausalito, CA, USA; ^2^Animal ScanRedwood City, CA, USA; ^3^Animal Internal Medicine and Specialty ServicesSan Francisco, CA, USA; ^4^Psychology Department, University of California at Santa CruzSanta Cruz, CA, USA; ^5^Department of Life Sciences, Texas A&M UniversityCorpus Christi, TX, USA

**Keywords:** *Zalophus*, gas bubble disease, diving mammals, brain MRI, veterinary medicine

## Abstract

A yearling California sea lion (*Zalophus californianus*) was admitted into rehabilitation with signs of cerebellar pathology. Diagnostic imaging that included radiography and magnetic resonance imaging (MRI) demonstrated space-occupying lesions predominantly in the cerebellum that were filled partially by CSF-like fluid and partially by gas, and cerebral lesions that were fluid filled. Over a maximum period of 4 months, the brain lesions reduced in size and the gas resorbed and was replaced by CSF-like fluid. In humans, the cerebellum is known to be essential for automating practiced movement patterns (e.g., learning to touch-type), also known as procedural learning or the consolidation of “motor memory.” To test the animal in this study for motor memory deficits, an alternation task in a two-choice maze was utilized. The sea lion performed poorly similar to another case of pneumocerebellum previously reported, and contrary to data acquired from a group of sea lions with specific hippocampal injury. The learning deficits were attributed to the cerebellar injury. These data provide important insight both to the clinical presentation and behavioral observations of cerebellar injury in sea lions, as well as providing an initial model for long-term outcome following cerebellar injury. The specific etiology of the gas could not be determined. The live status of the patient with recovery suggests that the most likely etiologies for the gas are either *de novo* formation or air emboli secondary to trauma. A small air gun pellet was present within and was removed from soft tissues adjacent to the tympanic bulla. While no evidence to support the pellet striking bone was found, altered dive pattern associated with this human interaction may have provided the opportunity for gas bubble formation to occur. The similarity in distribution of the gas bubble related lesions in this case compared with another previously published case of pneumocerebellum suggests that preferential perfusion of the brain, and more specifically the cerebellum, may occur during diving events.

## Introduction

Diving marine mammals have elaborate anatomical, physiological, and behavioral adaptations to reduce N_2_ gas loading although the detailed mechanics of gas kinetics is a contentious subject area of renewed and growing interest among scientists (Hooker et al., 2012). Previously it was presumed that diving adaptations including pulmonary shunting, bradycardia, and apnea protected marine mammals from supersaturation and associated gas bubble formation, but recent findings in stranded cetaceans where incidental gas accumulations have been identified, and hyperbaric CT chamber observations in cadavers, have disputed this view-point, suggesting that supersaturation of tissues and pathological gas bubble accumulations are possible (Moore et al., 2011; Dennison et al., 2012). Furthermore, a single case of pneumocerebellum in a California sea lion was recently described (Van Bonn et al., 2011). While this case has striking similarities to the previously published case, the clinical and behavioral assessments for motor learning that were possible combined with the survival of the animal provides valuable information for veterinarians, veterinary technicians, animal care specialists, rehabilitators, behavioralists, trainers, and physiologists on both gas bubble related pathology and space-occupying encephalopathies in sea lions in general. These data provided here will assist care-givers in the prognosis and expectations following marine mammal cerebellar injury. Furthermore the data raises questions about perfusion during dives, with apparent preferential perfusion to the cerebellum, as well as demonstrating the potential for gas bubble related pathologies when normal protective mechanisms are disrupted.

## Methods

A 25 kg body weight, 108 cm standard length (nose to tail tip), male, yearling California sea lion (*Zalophus californianus*) came ashore in apparent distress on 4 October 2011 near Morro Bay, CA, USA (35.361′N, −120.87′W). The animal was initially described as dull, in fair body condition but having difficulty moving. The animal was captured uneventfully and transported to The Marine Mammal Center (TMMC) rehabilitation hospital facilities in Sausalito, CA, USA. Starting at the first day of hospitalization the animal was fed and ate thawed, previously fresh-frozen whole fish, navigated the enclosure well and interacted with other inpatient animals as expected. An initial physical examination was conducted under manual restraint on 6 October 2011. Significant clinical findings at that time included a moderate ataxia suggesting proprioceptive deficits, lack of menace reflexes in both eyes, and auscultation with a stethoscope yielded subjectively increased respiratory sounds over both sides of the thorax.

Orthogonal survey radiographs of the skull and thorax were acquired on 6 October 2011. Standard complete blood counts and serum biochemical profiles were conducted following blood collection on 6 and 24 October 2011 and again on 15 February 2012. Initial treatment consisted of amoxicillin at 22 mg/kg by mouth twice daily, clindamycin at 5.5 mg/kg by mouth twice daily, 0.2 mg/kg ivermectin as a single dose by mouth and 4 mg/kg carprofen by mouth once daily and three intramuscular injections of 1 cc of vitamin B complex solution followed by routine oral vitamin supplementation in food fish in accordance with standard TMMC procedure.

The neurological deficits observed indicated a need for magnetic resonance imaging (MRI) evaluation and a small metallic pellet observed during radiographs was surgically removed under general anesthesia to permit acquisition of this study. Following pellet removal, two MRI studies of the brain were conducted under anesthesia with mechanical ventilation on 26 October 2011 and again on 15 February 2012. A Siemens Magnetom Symphony 1.5T magnet with an extremity coil was utilized for the studies (Siemens Medical Solutions USA, Inc., Malvern, PA, USA). Standard sequences appropriate for brain evaluation in veterinary species were acquired: T2W, T1W pre and post gadolinium (Gd) administration, FLAIR, T2*W, and PDW studies with image acquisition in the transverse plane (all sequences), dorsal plane (T1W post Gd only) and sagittal plane (T2W and T1W post Gd only). Additionally, oblique T2W and PDW transverse images were acquired for evaluation of the hippocampus (Montie et al., 2009). Gadolinium-based contrast medium (Omniscan, GE Healthcare, Inc., Princeton, NJ, USA) was administered prior to the post contrast series via intravenous injection into the subclavian vein at a dose of 0.01 mmol/kg.

A behavioral assessment of the animal’s motor learning was performed between 4 and 23 November, 2011 at the University of California Santa Cruz Long Marine Laboratory. In humans, the cerebellum is known to be essential for automating practiced movement patterns (e.g., learning to touch-type), also known as procedural learning or the consolidation of “motor memory” (Shadmehr and Holcomb, 1997). To test the animal in this study for motor memory deficits, an alternation task in a two-choice maze was utilized. Alternation learning in such mazes has been validated as a measure of motor learning (Wood et al., 2000; Dudchenko, 2012), and has a long history of use in mouse models of cerebellar damage (Petrosini et al., 1996; Lalonde and Strazielle, 2003). This particular instantiation was developed to assess animals with presumed or confirmed exposure to domoic acid, a naturally occurring harmful algal neurotoxin that often leads to hippocampal damage in exposed sea lions (Goldstein et al., 2008). The alternation task was used opportunistically here, both due to its being ideally suited for establishing a behavioral correlate to cerebellar damage, and due to the lack of standardized tests for marine mammals in veterinary practice. Because the current subject was tested under the same experimental conditions as the previous subjects (including location, time of day, reinforcement schedule, etc.), none of which had cerebellar lesions as verified by assessment of MRI, the data from these previous subjects was used as control data for comparison to this case.

The maze consisted of a approximately 3 m chute, open on one end and terminating at the other in two hinged doors, one on the right and one on the left. The animal was trained to navigate the chute repeatedly (one trip through the maze constituted a “trial”), each time exiting through either the right or the left door, and then returning to the beginning of the chute for another trial. If the animal selected the correct door, it received a small fish reward. If the animal selected the incorrect door, no reward was provided. In either case, the animal was to return to the beginning of the chute for another attempt. On the first trial of each experimental session the animal was rewarded regardless of which door it selected. On all following trials within the session, the correct door was defined as the door opposite from that most recently selected. Thus, to maximize reward, the animal needed to alternate doors on each subsequent trial, navigating the maze in a left, right, left, right, etc., pattern. As with previous subjects participating in this same alternation task as part of the previously mentioned domoic acid study, the animal was to continue the experiment until it had learned to alternate reliably, defined as making 17 or more correct door choices on two consecutive, 20-trial sessions (i.e., performing at or above 85%). As with previous subjects tested in this task, a variable number of trials (between 18 and 65) were conducted on each day, beginning no earlier than 10:00 a.m. and ending no later than 5:00 p.m.

## Results

### Labwork

Clinical hematology and serum chemistry results were unremarkable with the exception of a mild elevation in CPK (2827 U/L measured value; 80–1058 U/L reference values) on 6 October 2011 that had normalized by 24 October 2011, and a microfilaremia reported on 24 October 2011 and 15 February 2012. Microfilaremia due to the filarid nematode *Acanthocheilonema odenhali* is a common finding in California sea lions admitted to TMMC (Dailey, 2001).

### Diagnostic imaging

Survey radiographs of the skull demonstrated gas accumulation within the caudal fossa. A small metallic pellet was observed adjacent to the right tympanic bulla without evidence of osseous damage. There was no discernible change within the soft tissues surrounding the pellet within the limitations of radiography. Thoracic radiographs were within normal limits.

On the initial MRI, multifocal lesions were observed throughout the cerebrum and cerebellum.

**Figure 1:**
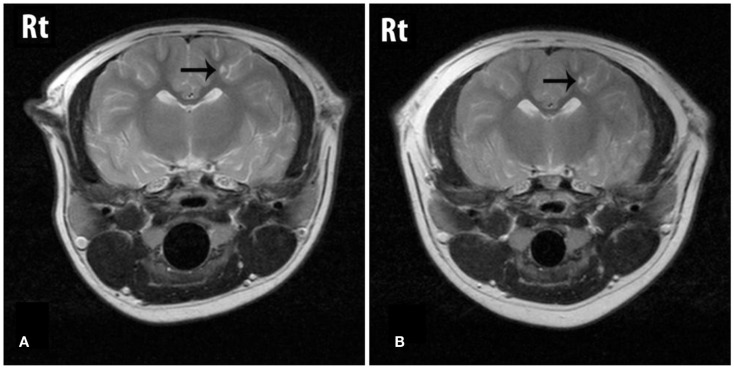
**T2W transverse images through the cerebrum showing a lesion (arrow) that is fluid filled on both the initial (A) and the follow-up (B) studies**. The lesion is considered static between dates.

**Figure 2:**
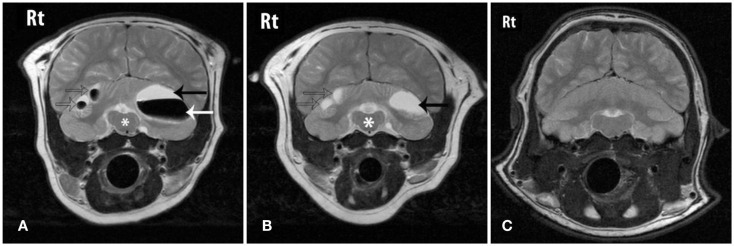
**Transverse T2W images through the caudal fossa of the patient on initial MRI (A), follow-up MRI (B) and a grossly normal cerebellum from another sea lion for comparison (C)**. “Rt” represents the animal’s right-side. **(A)** Three cerebellar lesions are shown (arrows) that contain fluid that is CSF-like in character (black arrow) and gas (white arrow). The mass effect of the lesions is causing distortion of the brainstem (*). **(B)** On follow-up MRI the lesions (arrows) shown in **(A)** have reduced in size, normalizing the appearance of the brainstem (*). The previously seen gas has been resorbed and the lesions now contain only fluid. **(C)** An image at the same level from a normal sea lion at the same level.

The cerebral lesions were smaller than the cerebellar lesions, ranging from 3 mm × 1 mm in size to 7 mm × 4 mm, and all were filled with CSF-like fluid, consistent with encephalomalacia (Figure 1). The multiple cerebellar lesions ranged in size from 0.7 cm × 1.3 cm to 3 cm × 4 cm in size. The cerebellar lesions contained both gravitationally dependent CSF-like fluid and antigravitational gas accumulations consistent with pneumatocele lesions converting to encephalomalacia. The peripheral brain parenchyma surrounding the lesions was contrast-enhancing post Gd administration. A mass effect was present causing rightward displacement of the falx and compression of the fourth ventricle. Mild bilateral lateral and fourth ventriculomegaly were also observed and presumed to be due to obstruction secondary to the mass effects of the lesions (Figure 2).

On follow-up MRI contrast enhancement of the lesion peripheries was no longer identified. The cerebral and cerebellar lesions seen previously all persisted, and no new lesions were identified. All lesions that had previously contained a gas component were now filled with CSF-like fluid and the size of those lesions had reduced, resulting in resolution of the mass effect and resolution of the ventriculomegaly. The smaller cerebral and cerebellar lesions that initially were filled with CSF-like fluid were static in character and size (Figures 1 and 2).

### Behavioral testing of motor learning

This animal demonstrated remarkably similar deficits in performance on the maze task as the previously published animal with pneumocerebellum, which had taken 696 trials to meet the criterion of two consecutive sessions at or above 85%. After 3 weeks of testing, the animal in the current study could no longer be housed at Long Marine Lab, so testing ceased. At this point, the animal had completed 632 trips through the maze and had still not met the alternation criterion. However, the number of correct door choices on the last two 20-trial sessions was 18 and 16, respectively; suggesting general facility with alternation had been acquired. The sea lion completed 632 trials, which was 1.87 standard deviations (139.4) greater than the mean number of trials required to achieve the criterion (372) that was established by 10 other California sea lions that previously participated in the same testing. None of these previous animals required a number of trials more than one standard deviation above the mean.

## Discussion

The lesions in the cerebellum were strikingly similar to those documented in our initial case (Van Bonn et al., 2011). This animal demonstrated remarkably similar deficits in performance on the maze task as the animal from the initial case study, requiring markedly more trials to learn the alternation task than the previous sea lions tested. The cerebellum was normal on MRI in these 10 previous sea lions. Five of these animals had brain damage specific to hippocampal formation, likely as a result of toxic exposure to domoic acid, while the other five appeared to have healthy brains without diagnosable lesions.

The exact function of the hippocampus is still debated, but it is broadly agreed that it does not subserve procedural memory or motor learning (Squire, 1992). Alternation learning is a long-standing, validated approach to demonstrating motor learning deficits in animals with cerebellar lesions – in our case it had the added benefit of allowing use of a pre-existing databank of matched control results. While it is true that the exact function of the hippocampus in humans and non-humans is debated – as it likewise continues to be for most brain areas – the idea that the hippocampus is primarily responsible for committing short-term memory to long-term memory is outmoded.

Newer neurophysiological models strongly indicate the hippocampus binds together disparate sensory streams into compound representations, regardless of time-scale, subserving episodic representation both in working memory and long-term memory. In fact, hippocampal damage has essentially no impact on alternation learning (which might, mistakenly, be viewed as primarily a working memory task), which, again, is subserved by motor learning areas such as the cerebellum and basal ganglia. Rather, it is with forced delay alternation that the impairment emerges with hippocampal damage. Because basic alternation can be acquired via motor learning, performance between animals with and without hippocampal damage should not differ on acquisition – rather, animals with hippocampal damage show differential impairment when a delay is enforced before each trial of the alternation task (e.g., Wood et al., 2000). So, given the matched testing condition, the prior animals, both those with hippocampal damage and those presenting as neurologically “normal,” can serve as controls for the performance of the current and previous subject with cerebellum damage. Indeed, there are no inter-group differences in acquisition on the basic alternation between the animals with and without hippocampal damage.

The fact that focal hippocampal damage does not impair learning, but focal cerebellum damage does, strongly suggests a functional dissociation – many cognitive capabilities can be disrupted by damage to a wide range of brain areas, so this type of dissociation is actually quite important in demonstrating valid structure-function linkages, particularly given a small sample size as here. Double dissociations are an essential tool in establishing linkages between structure and function in the cognitive neurosciences (Teuber, 1955; Fodor, 1983), indicating that observed deficits in behavior are due to damage to specific brain areas as opposed to being general products of any disruption in the brain. Only the two animals with cerebellum damage represent a different population in regards to trials to acquisition. The present results strongly indicate a sizable impairment in motor learning in the current subject as a result of cerebellar lesions.

The cranium imaging findings radiographically and on MRI were almost identical to the prior case, although cerebral as well as cerebellar lesions were identified in this current case on MRI. Perhaps more importantly, the similar location of the gas bubble accumulations within the cerebellar tissues, regardless of the origin of the gas, may confirm selective perfusion of the brain during dives. The space-occupying lesions within the cerebellum were partially CSF-like fluid and partially gas filled on the initial MRI study. Based on the progression of lesions seen in the first published case (Van Bonn et al., 2011), the mix of CSF fluid and gas within the lesions was thought to represent resorption of the gas and filling of the residual spaces with CSF.

The etiology of the gas remains uncertain. Gas bubbles may, hypothetically, form *de novo* in supersaturated states, may be introduced iatrogenically, can represent gas-producing bacterial infection or decomposition, or occur due to trauma permitting entry of gas into the circulation. Contrary to the previous case we reported, there was no evidence of rib fractures or other trauma in the present case to account for gas bubbles entering the circulation. The recovery of the patient given the location of the gas makes a gas-producing bacterial infection very unlikely, and the live status of the patient rules out decomposition as a cause. The air gun pellet observed on the initial survey radiographs was not deformed on removal, nor was there any radiographic evidence that it impacted any bony structures. It is possible that this foreign body caused microscopic trauma to the tympanic bulla, tympanic cavity, auditory tube, or external acoustic meatus that may have resulted in direct air leakage intracranially or via the circulation; however, there was no gross evidence of this on any of the imaging studies. Despite the lack of evidence of trauma caused by the pellet, the harassment of a gunshot is expected to have caused a behavioral change in the animal with speculated effects including alteration in dive time, rate of ascent or descent or other feature that could contribute to *de novo* gas bubble formation if the sea lion was in a supersaturated state.

The most realistic etiologies for the gas are trauma resulting in air emboli, or *de novo* formation from supersaturation. If gas bubbles were introduced into the circulation, following Boyle’s Law, simple physics predicts that compressing a gas volume the size of the larger lesions in this animal at presentation to a volume approximating a sea lion erythrocyte (approximately 100 fl) would require approximately 100,000,000 atmospheres of pressure, equivalent to water pressure at an ocean depth of 10,000,000 m. This is obviously impossible and negates the assumption that a gas bubble of a size that would allow it to circulate within vascular spaces simply got lodged in the brain during a deep dive and expanded during ascent.

In the absence of evidence for an underlying cause, *de novo* formation of gas bubbles has to be considered. In the human diving literature, several studies suggest the presence of gas seeds or nuclei as the cause of bubble formation in decompression injury (Vann, 1989). A number of different models have been proposed in attempt to explain how these nuclei persist. The permeability model suggests that the nuclei are stabilized by adsorbed amphiphilic molecules, while the crevice model proposes that the nuclei are trapped and stabilized in hydrophobic crevices. Other mechanisms include hydrodynamic and mechanical effects that cause coalescence and growth, e.g., rectified diffusion and tribonucelation (Tikuisis and Gerth, 2003). However, bubble growth, even from a pre-existing nucleus, requires that the dissolved gas tension exceeds the gas partial pressure in the bubble. A growing body of evidence from bone lesions in sperm whale bones; stranded, bycaught, and live odontocetes and pinnipeds does suggest that marine mammals often experience blood and tissue tensions that may result in the formation of bubbles (Moore and Early, 2004; Moore et al., 2009; Dennison et al., 2012; de Quirós et al., 2012).

In the previous case we reported, the animal was fitted with a satellite tag capable of recording spatial movement and dive depths. The animal did range and dive as expected but restranded after losing considerable body weight and ultimately died from pleuropneumonia. It is unknown if the brain damage, the thoracic pathology, or the combination of pathologies in that case resulted in failure to successfully forage despite normal dive range and depth recordings, however the possibility of that animal’s demise being at least in part due to the brain damage raised questions about this second animal’s ability to survive on release. As with the previous subject, the current subject demonstrated a marked deficit in motor learning capability, which has been shown to be critical to navigation (Rondi-Reig and Burguiere, 2005) and reward-based learning (Thoma et al., 2008) in the laboratory. Given the neurological deficits seen clinically and documented during motor testing, in combination with the outcome of the prior case, the decision was made that this animal was not a release candidate. It was subsequently placed in a captive-maintained environment, and as of the date of publication is thriving in that setting.

## Conflict of Interest Statement

The authors declare that the research was conducted in the absence of any commercial or financial relationships that could be construed as a potential conflict of interest.
